# Spasmodic Action of the Dura Mater

**Published:** 1834

**Authors:** 


### X.—Wound of the Longitudinal Sinus and Brain—Convulsions—Spasmodic Contraction of the Dura Mater.

We are indebted to Dr. John F. Carmichael, of Mississippi, formerly a surgeon in the army of Gen. Wayne, for the following fact.

A soldier in the celebrated battle of the 20th of August, 1794, was shot through the head. The ball entered the centre of the upper part of the forehead, and passing between the tables of the skull, made its exit about three inches back. The bone was of course torn to pieces, and a great number of fragments were driven downwards, even to the depth of two inches into the brain, a considerable quantity of which escaped. The symptoms were those of apoplexy. Upon removing the scalp and trimming off the edges of the bone, the oblong opening of the cranium, was wide enough to admit the finger. No hœmorrhage *then* occurred, but was said to have been profuse before the patient came into Dr. Carmichael's hands. In attempting to extract the spiculæ of bone, with forceps pushed into the substance of the brain, a kind of cataleptic convulsion came on, and was repeated at each operation, till the whole were extracted. *When the first convulsion took place, the doctor had his finger in the brain, feeling for one of the fragments, and was surprised to find that the lacerated dura mater closely contracted about it, till the spasm ceased, when the membrane simultaneously relaxed: subsequently, on introducing the forceps, the same contraction several times recurred so as to grasp the instrument, coming and going with the general spasms.* All the spiculæ being removed, the wound was dressed in the usual manner, and the patient speedily recovered. The professional and personal respectability of Dr. C., do not permit us to doubt the accuracy of this observation, which appears to be sufficiently curious to merit publication, and which was witnessed by many intelligent officers of the army.

